# The Role of Diffusion-Weighted Imaging in Characterizing Benign and Malignant Breast Lesions: A Retrospective Study

**DOI:** 10.7759/cureus.66472

**Published:** 2024-08-08

**Authors:** Stany Jerosha, Sakthi Ganesh Subramonian, Arunkumar Mohanakrishnan, Karthik Krishna Ramakrishnan, Paarthipan Natarajan

**Affiliations:** 1 Radiodiagnosis, Saveetha Medical College and Hospitals, Saveetha Institute of Medical and Technical Sciences, Saveetha University, Chennai, IND

**Keywords:** bi-rads, apparent diffusion coefficient (adc), screening of breast cancer, mri breast, breast lesion

## Abstract

Introduction

Diffusion-weighted imaging (DWI) is a promising magnetic resonance imaging (MRI) technique for differentiating between benign and malignant breast lesions. This study set out to assess the diagnostic utility of DWI and apparent diffusion coefficient (ADC) values in the characterization of breast lesions.

Materials and methods

A retrospective analysis comprised 30 patients with breast lesions who had breast MRI with DWI. The histopathological findings, ADC readings, and conventional MRI features were all analyzed. The receiver operating characteristic (ROC) curve analysis method was utilized to assess the diagnostic accuracy of DWI.

Results

Out of the 30 lesions, 22 (73.3%) were benign and eight (26.7%) were malignant. Malignant lesions exhibited significantly lower ADC values (p < 0.001) compared to benign lesions. An ADC cutoff value of 1.1 × 10^-3^ mm^2^/s was optimal for differentiating benign from malignant lesions, yielding 90.81% sensitivity, 91.51% specificity, and 91.5% accuracy.

Conclusion

Combining DWI with quantitative ADC analysis is a helpful, non-invasive method for the characterization of breast lesions. It shows excellent diagnostic accuracy in identifying benign and malignant lesions, which may cut down on pointless biopsies and help with patient management.

## Introduction

Breast cancer is a leading cause of cancer-related mortality among women worldwide [1]. Early detection and accurate characterization of breast lesions are crucial for effective treatment and improved patient outcomes. However, traditional imaging techniques, such as mammography and ultrasound, have limitations in distinguishing between benign and malignant lesions [2]. Magnetic resonance imaging (MRI), with its multiparametric capabilities and superior soft tissue contrast, has become an essential tool for evaluating breast lesions [3].

Diffusion-weighted imaging (DWI), a functional MRI technique, measures the random movement of water molecules within tissues [4]. The apparent diffusion coefficient (ADC), derived from DWI, indicates the extent of water molecule diffusion restriction, which is influenced by tissue microstructure and cellularity [5]. Malignant breast lesions, due to their higher cellularity and restricted diffusion, typically exhibit lower ADC values compared to benign lesions [6,7]. Numerous studies have demonstrated the role of DWI in characterizing breast lesions, highlighting its ability to improve diagnostic accuracy and reduce unnecessary biopsies [8-10].

This study aims to assess the diagnostic efficacy of DWI and ADC values in differentiating benign from malignant breast lesions and to determine the optimal ADC cutoff value for lesion characterization.

## Materials and methods

The Department of Radiology at Saveetha Medical College and Hospitals conducted a retrospective study involving 30 patients with breast lesions who underwent breast MRI with DWI between March 2023 and July 2023. The inclusion criteria were: (I) patients with suspicious breast lesions detected by other imaging modalities, such as ultrasound or mammography; (II) patients with confirmed pathology of the breast lesion through biopsy or surgical excision; and (III) patients without contraindications for MRI. The exclusion criteria included: (I) pregnancy in the first trimester; (II) severe claustrophobia; (III) the presence of aneurysm clips; (IV) cardiac pacemakers; and (V) unsafe metallic implants.

The MRI procedure utilized an eight-channel dedicated breast coil with a Philips Multiva 1.5 Tesla MRI scanner (Philips, Amsterdam, Netherlands). The imaging protocol included: (I) a three-plane localizer; (II) DWI with ADC mapping; (III) axial and sagittal T2-weighted imaging; (IV) axial and sagittal T2 spectral attenuated inversion recovery (SPAIR); and (V) contrast-enhanced imaging (eTHRIVE T1 sagittal, T1 spectral pre-saturation with inversion recovery (SPIR) axial, and sagittal) when clinically warranted. DWI was acquired in the axial plane using a single-shot echo-planar imaging sequence (b-values of 0 and 1000 s/mm²; repetition time (TR)/echo time (TE) effective range: 1000/74 ms; slice thickness: 5 mm; field of view (FOV): 30 x 30 x 25 cm; matrix: 384 x 256) before administering contrast.

**Figure 1:**
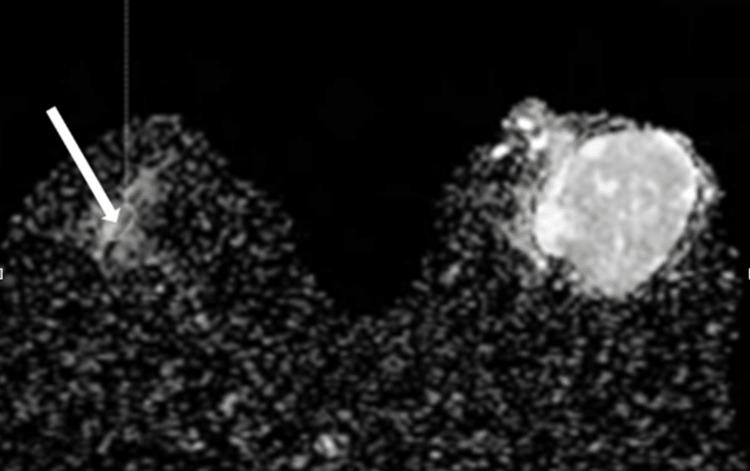
ADC map of breast ADC (apparent diffusion coefficient) map of the breast showing the placement of region of interest in areas showing true diffusion restriction (long white arrow), avoiding areas of hemorrhage and necrosis.

T2 signal intensity and other conventional MRI features were assessed. To measure ADC values, regions of interest (ROIs) were placed on ADC maps in areas showing true diffusion restriction, avoiding necrosis and bleeding (Figure 1).

After placing two ROIs measuring 50 ± 10 mm², the mean ADC value was calculated. The ADC values and DWI results were compared to the findings from conventional MRI.

The histopathological diagnosis of breast lesions, obtained through biopsy or surgical excision, served as the gold standard for assessing the diagnostic effectiveness of DWI. The statistical analysis was performed using IBM SPSS Statistics for Windows, Version 25 (Released 2017; IBM Corp., Armonk, NY, USA). The Mann-Whitney U test was used to compare the ADC values of benign and malignant lesions. ROC curve analysis was conducted to determine the optimal ADC cutoff value for distinguishing between benign and malignant lesions. The calculated metrics included likelihood ratios, sensitivity, specificity, positive and negative likelihood ratios, and accuracy. A p-value of less than 0.05 was considered statistically significant.

## Results

A total of 30 patients with 30 breast lesions (mean age: 51.8 years; range: 30-70 years) were included in the study. The clinical indications for breast MRI were: breast lump (n = 11, or 36.7%); breast pain (n = 9, or 30%); nipple discharge (n = 7, or 23.3%); and screening (n = 3, or 10%) (Table 1).

**Table 1:** Clinical indications for breast MRI MRI: Magnetic resonance imaging

Clinical indications	Number	Percentage (%)
Screening	3	10
Pain	9	30
Lump	11	36.7
Nipple discharge	7	23.3

Typical characteristics of an MRI showed that 22 lesions (73.3%) had high signal intensity on T2-weighted conventional MRI, whereas eight lesions (26.7%) had low signal intensity (Table 2).

**Table 2:** Signal intensities of the lesions

Signal of lesion	Number	Percentage (%)
High	22	73.3
Low	8	26.7

Analysis of DWI and ADC for benign lesions revealed a mean ADC reading of 1.41 ± 0.56 × 10^-3^ mm^2^/s, while for malignant lesions, it was 0.89 ± 0.18 × 10^-3^ mm^2^/s. When compared with benign lesions, malignant lesions exhibited significantly lower ADC values (p < 0.001) (Table 3).

**Table 3:** ADC values of benign and malignant breast lesions The statistical test used in the study is the Mann-Whitney U test. A p-value less than 0.05 was considered statistically significant. Analysis of DWI and ADC for benign lesions, the mean ADC readings were 1.41 ± 0.56 × 10^-3^ mm^2^/s, and for malignant lesions, they were 0.89 ± 0.18 × 10^-3^ mm^2^/s. ADC: Apparent diffusion coefficient; DWI: Diffusion-weighted imaging

Histopathological diagnosis	ADC value (range) (×10^-3^ mm^2^/s)
Malignant lesion (M)	0.69-1.20
Benign lesions (cystic and solid) (B)	0.65-2.4

By utilizing ROC curve analysis, the ideal ADC cutoff value for distinguishing between benign and malignant lesions was found to be 1.1 × 10^-3^ mm^2^/s. The results revealed that the positive likelihood ratio was 10.60, the negative likelihood ratio was 0.09, the accuracy was 91.5%, the sensitivity was 90.81%, and the specificity was 91.51% (Table 4).

**Table 4:** Diagnostic efficacy of the optimal ADC cutoff value ADC: Apparent diffusion coefficient

Parameters	Benign (%)	Malignancy (%)
Sensitivity	91.35	90.81
Specificity	90.81	91.51
Positive likelihood ratio	10.06	10.60
Negative likelihood ratio	0.09	0.09
Accuracy	91.5	91.5

The histopathological analysis revealed 22 benign lesions (73.3%) and eight malignant lesions (26.7%). The malignant lesions were invasive carcinomas of any kind, while the benign lesions included fibroadenomas, breast cysts, and benign stromal myxoid lesions (Table 5).

**Table 5:** Histopathological diagnosis of breast lesions FNAC: Fine needle aspiration cytology; HPE: Histopathological examination

FNAC/HPE	Number	Percentage (%)
Benign	22	73.3
Malignant	8	26.7

## Discussion

Our study demonstrates the accuracy of DWI in differentiating between benign and malignant breast lesions. Consistent with previous research, our data show that malignant lesions have significantly lower ADC values than benign lesions [6,7,11]. This can be attributed to the fact that malignant tissues have restricted diffusion and higher cellularity [12].

Our findings revealed excellent sensitivity, specificity, and accuracy in distinguishing benign from malignant lesions using the optimal ADC cutoff value of 1.1 × 10^-3^ mm^2^/s. This result aligns with other studies that reported cutoff values ranging from 0.9 to 1.3 × 10^-3^ mm^2^/s [8,13,14]. The high diagnostic accuracy of DWI underscores its potential to reduce unnecessary biopsies in patients with benign lesions and to assist in the early detection of malignancies.

In addition, we evaluated conventional MRI features, such as T2 signal intensity. On T2-weighted images, most benign lesions exhibited high signal intensities, while most malignant lesions showed low signal intensities. This observation is consistent with the literature, as benign lesions typically have higher water content and longer T2 relaxation times compared to malignant lesions [15,16].

Integrating DWI into the breast MRI protocol provides complementary information to traditional MRI sequences. The combination of morphological and functional data enhances diagnostic accuracy and confidence in lesion characterization [17,18]. Furthermore, DWI can be advantageous for patients who should not receive contrast media, as it is a non-invasive procedure that does not require the administration of contrast agents [19].

However, our study has some limitations. The small sample size and retrospective design may limit the generalizability of the results. Additionally, potential bias may have been introduced since only one radiologist performed the ADC measurements. Future research with larger sample sizes and multiple readers is necessary to validate these findings.

In summary, DWI combined with quantitative ADC analysis is a valuable non-invasive method for identifying breast lesions. With an ideal ADC cutoff value of 1.1 × 10^-3^ mm^2^/s, it demonstrates high diagnostic efficiency in distinguishing between benign and malignant lesions. Incorporating DWI into the breast MRI protocol can improve lesion characterization, reduce unnecessary biopsies, and provide valuable guidance for patient treatment. However, further studies are needed to confirm the clinical utility of DWI in breast imaging.

In the last few years, technological developments in the surgical field have been rapid and are continuously evolving. One of the most revolutionary breakthroughs was the introduction of the IoT concept within surgical practice.

The Internet of Things (IoT) plays a significant role in characterizing benign and malignant breast lesions by enabling advanced diagnostic and monitoring capabilities. IoT integrates various smart devices and sensors to collect, process, and analyze data in real time. For example, connected imaging modalities like mammography, MRI, and ultrasound can transmit data to a central system for comprehensive analysis. This connectivity allows for the aggregation of imaging data, patient history, and other relevant information, facilitating more accurate differentiation between benign and malignant lesions. Additionally, IoT-based systems can support continuous monitoring of patients, enabling early detection of changes in breast tissue that may indicate malignancy. The use of IoT in this context enhances diagnostic precision, supports personalized treatment planning, and may improve patient outcomes by facilitating timely interventions [20].

This approach aligns with the broader concept of the Internet of Surgical Things (IoST), which includes the application of IoT technologies in various aspects of surgical practice, such as imaging and patient monitoring [20]. The incorporation of IoT in medical practice, specifically in breast lesion characterization, represents a paradigm shift towards more integrated and intelligent healthcare systems.

Limitations 

The study on the role of DWI in characterizing benign and malignant breast lesions has certain limitations. First, the small sample size and retrospective design may limit the generalizability of the findings. A larger sample size and prospective studies would be beneficial for validating these results. Second, the measurements of the ADC were conducted by a single radiologist, which could introduce measurement bias. Future studies should include multiple readers to ensure the reliability of ADC measurements. Additionally, while the study utilized a specific ADC cutoff value, the variability in cutoff values reported in the literature suggests that a standardized threshold may not be universally applicable. Further research is needed to establish consistent ADC cutoff values and to explore the clinical applicability of DWI in a broader range of patient populations.

## Conclusions

In conclusion, DWI combined with quantitative ADC analysis demonstrates excellent diagnostic accuracy in distinguishing between benign and malignant breast lesions. The optimal ADC cutoff value of 1.1 × 10^-3^ mm^2^/s can aid in clinical decision-making and lesion characterization. Integrating DWI into the breast MRI protocol offers valuable functional information that complements conventional MRI sequences. The non-invasive nature of DWI and its potential to reduce unnecessary biopsies underscore its value in breast imaging. However, to confirm these findings and establish the clinical utility of DWI in breast lesion characterization, further extensive research is required.
